# Supplementary material for: Identification of photoperiod-induced specific miRNAs in the adrenal glands of Sunite sheep (Ovis aries)
Source: Front Vet Sci. 2022 Jul 22;9:888207. doi: 10.3389/fvets.2022.888207 (PMC9354845; doi:10.3389/fvets.2022.888207)
Supplement: Supplementary file 1 [file Data_Sheet_1.ZIP › Supplementary file/miRDeep2 file.zip!/miRDeep2 file/Know_Structure/SPLP42A3/oar-mir-382.pdf]

miRBase precursor : oar-mir-382
Total read count : 3140
oar-miR-382-5p read count : 1769
oar-miR-382-3p read count : 1359
remaining reads : 12

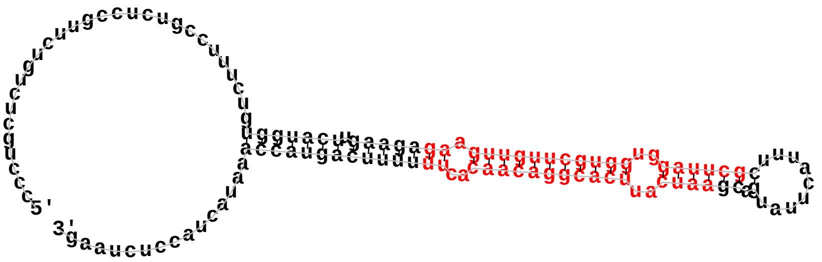

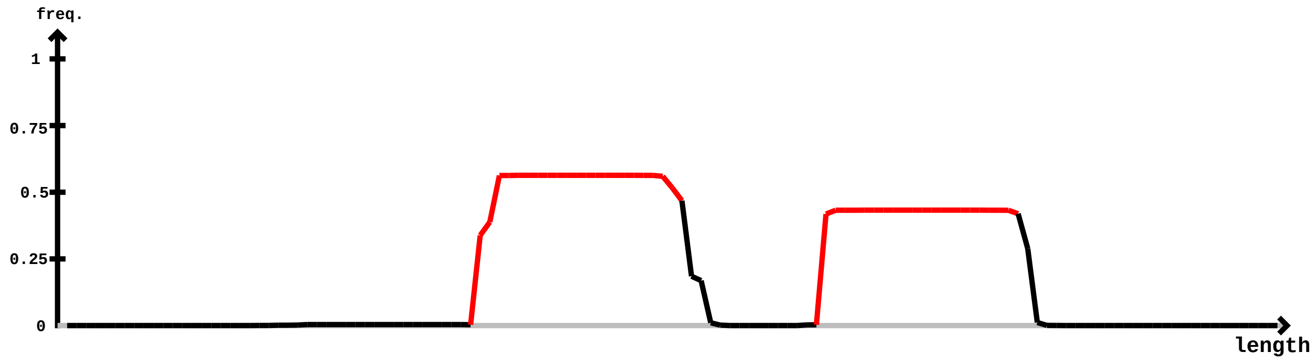

oar-miR-382-3p

oar-miR-382-5p

5'- cccugcucugucuugccucugccuuucugugguacuugaagagaaguuguucgugguggauucgcuuuacuuaugacgaaucauucacggacaacacuuuuuucaguaccaaauacuaccucuaag -3'	exp	
....((........)).............(((((((.(((((((.(((((((((((..(((((((.........).))))))..)))))))))))..)))))))))))))))...............	reads	mm
...................ugccuuucugugguacuugaaga..	1	0
.....................ccuuucugugguacuugaag...	2	0
.....................ccuuucugugguacuugaaga..	1	0
.......................uuucugugguacuugaaga..	2	0
........................uucugugguacuugaaga..	6	0
....gaaguuguucguggugg...	1	0
....gaaguuguucgugguggaG...	1	1
....gaaguuguucgugguggau...	10	0
....gaaguuguucgugguggauu..	134	0
....gaaguuguucgugguggauuc...	140	0
....gaaguuguucgugguggauucg..	749	0
....gaaguuguucgugguggauucgc...	26	0
....gaaguuguucgugguggauucgcu..	1	0
....aaguuguucgugguggauu..	2	0
....aaguuguucgugguggauuc...	11	0
....aaguuguucgugguggauucg..	136	0
....aaguuguucgugguggauucgc...	7	0
....aaguuguucgugguggauucgcu..	1	0
....aguuguucgugguggauucg..	4	0
....aguuguucgugguggauucgc...	19	0
....aguuguucgugguggauucgcu..	494	0
....aguuguucgugguggauucgcuu...	28	0
....aguuguucgugguggauucgcuuu..	3	0
....uuguucgugguggauucgcuuu..	2	0
....cgaaucauucacggacaac...............................	1	0
....cgaaucauucacggacaacacu............................	3	0
....cgaaucauucacggacaacacuu...........................	4	0
....gaaucauucacggacaacacuuu..........................	1	0
....aaucauucacggacaacac.............................	1	0
....aaucauucacggacaacacu............................	37	0
....aaucauucacggacaacacuu...........................	403	0
....aaucauucacggacaacacuuu..........................	843	0
....aaucauucacggacaacacuuuu.........................	20	0
....aucauucacggacaacacuu...........................	3	0

```
                                                                                  oar-miR-382-3p
                                            oar-miR-382-5p
cccugcucugucuugccucugccuuucugugguacuugaagagaaguuguucgugguggauucgcuuuacuuaugacgaaucauucacggacaacacuuuuuucaguaccaaauacuaccucuaag

..............................................................................aucauucacggacaacacuuu.........................    26    0
..............................................................................aucauucacggacaacacuuuu........................    14    0
..............................................................................aucauucacggacaacacuuuuu.......................    1     0
..............................................................................aucauucacggacaacacuuuuuu......................    1     0
.................................................................................auucacggacaacacuuu.........................    1     0
```
